# A Conterminous USA-Scale Map of Relative Tidal Marsh Elevation

**DOI:** 10.1007/s12237-021-01027-9

**Published:** 2022-01-12

**Authors:** James R. Holmquist, Lisamarie Windham-Myers

**Affiliations:** 1 Smithsonian Environmental Research Center, 647 Contees Wharf Road, Edgewater, MD 21037 USA; 2 U.S. Geological Survey, Water Mission Area, 345 Middlefield Road, Menlo Park, CA 94025 USA

**Keywords:** Saltmarsh, Coastal wetland, Elevation, LiDAR, Mapping, Tidal flooding

## Abstract

Tidal wetlands provide myriad ecosystem services across local to global scales. With their uncertain vulnerability or resilience to rising sea levels, there is a need for mapping flooding drivers and vulnerability proxies for these ecosystems at a national scale. However, tidal wetlands in the conterminous USA are diverse with differing elevation gradients, and tidal amplitudes, making broad geographic comparisons difficult. To address this, a national-scale map of relative tidal elevation (Z*_MHW_), a physical metric that normalizes elevation to tidal amplitude at mean high water (MHW), was constructed for the first time at 30 × 30-m resolution spanning the conterminous USA. Contrary to two study hypotheses, watershed-level median Z*_MHW_ and its variability generally increased from north to south as a function of tidal amplitude and relative sea-level rise. These trends were also observed in a reanalysis of ground elevation data from the Pacific Coast by Janousek et al. (Estuaries and Coasts 42 (1): 85–98, 2019). Supporting a third hypothesis, propagated uncertainty in Z*_MHW_ increased from north to south as light detection and ranging (LiDAR) errors had an outsized effect under narrowing tidal amplitudes. The drivers of Z*_MHW_ and its variability are difficult to determine because several potential causal variables are correlated with latitude, but future studies could investigate highest astronomical tide and diurnal high tide inequality as drivers of median Z*_MHW_ and Z*_MHW_ variability, respectively. Watersheds of the Gulf Coast often had propagated Z*_MHW_ uncertainty greater than the tidal amplitude itself emphasizing the diminished practicality of applying Z*_MHW_ as a flooding proxy to microtidal wetlands. Future studies could focus on validating and improving these physical map products and using them for synoptic modeling of tidal wetland carbon dynamics and sea-level rise vulnerability analyses.

**Supplementary Information:**

The online version contains supplementary material available at 10.1007/s12237-021-01027-9.

## Introduction

Tidal wetlands have the capacity to maintain an adaptive resilience to sea-level rise. As sea level increases, inundation drives elevation change by stimulating belowground biomass input and increasing the availability of sediment which can be trapped and deposited (Morris et al. 2002; Kirwan et al. 2013; Kirwan et al. 2016). Coastal wetlands of all land cover classes — saline to fresh, and woody, emergent, or submerged vegetation — accrete via these vegetative and inorganic soil formation pathways. However, resilience to sea-level rise is not assured or infinite because biological productivity and preservation are limited theoretically by plants’ abilities to fix carbon, and practically by ecological and physical constraints (Morris et al. 2016). Suspended sediment concentration can vary spatially because of a watershed slope, erodibility, size, and precipitation (Weston 2014), and temporally because of storms and upstream damming. Local rates of relative sea-level rise (RSLR), which take into account both eustatic and isostatic sea-level change, can vary greatly (Jankowski et al. 2017; Horton et al. 2018).

The conterminous USA (CONUS; Table 1) exhibits a range of physical conditions across its three coasts with tidal amplitude generally increasing from south to north, and being more muted in bays than in open water. RSLR, as measured over decades to centuries by long-term tide gauges, follows a similar pattern but is influenced by local drivers, and generally increases from north to south. Many studies have focused on drivers and processes controlling resiliency on local scales and regional scales (e.g., Thorne et al. 2018). However, there is a need for simple top-down metrics that can be used as resiliency proxies to aid in national-scale planning.

**Table 1:** A glossary of abbreviations used in this text

Term	Abbreviation
Aikake’s information criterion for small sample sizes	AICc
Coastal Change Analysis Program	C-CAP
Coastal Carbon Research Coordination Network	CCRCN
Coastal Elevation National Database	CoNED
Coastwide Reference Monitoring System	CRMS
Conterminous USA	CONUS
Digital elevation model	DEM
Diurnal high tide inequality	DHQ
Diurnal high tide inequality normalized by tidal amplitude at MHW	DHQ*_MHW_
Elevation	Z
Elevation normalized to tidal amplitude	Z*
Elevation normalized to tidal amplitude at mean high water	Z*_MHW_
Highest astronomical tide	HAT
Highest astronomical tide normalized to tidal amplitude at mean high water	HAT*_MHW_
Highest observed tide	HOT
Highest observed tide normalized to tidal amplitude at mean high water	HOT*_MHW_
Hydrologic unit code level-8	HUC8
Light detection and ranging	LiDAR
Marsh Equilibrium Model	MEM
Marsh Resilience to Sea-Level Rise	MARS
Mean high water	MHW
Mean higher high water	MHHW
Mean higher gigh water for spring tides	MHHWS
Mean sea level	MSL
Million hectares	M ha
NASA Carbon Monitoring System	NASA CMS
National Oceanic and Atmospheric Association	NOAA
National Wetlands Inventory	NWI
North American vertical datum of 1988	NAVD88
Relative sea-level rise	RSLR
Shuttle radar topography mission	SRTM
US Department of Agriculture-Natural Resources Conservation Service	USDA-NRCS
US Geological Survey	USGS
Wetland Accretion Rate Model of Ecosystem Resilience	WARMER

A fundamental aspect of assessing wetland structure and vulnerability is its relative elevation. Ganju et al. (2019) showed that in several well-studied sites across the USA, relative tidal elevation, especially elevation relative to mean high water, correlates with a different top-down marsh vulnerability metric, the unvegetated to vegetated area ratio. Marshes that are relatively low in the tidal frame may be in some stage of collapse and vegetation loss. The marsh resilience to sea-level rise index (also known as MARS index; Raposa et al. 2016) incorporates elevation as well as tidal range into its ranking, with lower indices of resilience for microtidal marshes and marshes lower in the tidal frame, and higher indices for macrotidal marshes and marshes higher in the tidal range. Elevation normalized to tidal amplitude (Z*) has been shown to correlate with carbon stocks in the Pacific Northwest (Peck et al. 2020). Despite increasing recognition by the coastal wetland community of the need to report relative elevations as metadata for observations, hydrologic settings are most commonly reported qualitatively as either “high” or “low” marsh environments, or by using indicative vegetation communities (e.g., short and tall *Spartina alterniflora*).

Z* is a dimensionless and functionally important variable used in models of marsh resiliency to sea-level rise. For example, both the marsh equilibrium model (MEM; Morris et al. 2002) and the Wetland Accretion Rate Model of Ecosystem Resilience (WARMER) (Swanson et al. 2014) use it to constrain relationships between plant productivity and flooding. It has the advantage of being simple to calculate and update, making sites across geographies more easily intercomparable in their physical interaction with tides.

Since uncertainty propagation is a vital part of monitoring and decision support (Dietze et al. 2018), we outline the sources of uncertainty in the mapped components making up Z*. Airborne light detection and ranging (LiDAR) data have the potential to generate high-resolution digital elevation models (DEMs) for mapping flood potential and are an important part of coastal wetland monitoring (Chmura 2013). However, they are often built to accuracy specifications relevant to assessing potential property damages (ASPRS 2004; Coveney 2013); coastal wetland processes are sensitive to centimeter-scale gradients and usually covered by thick vegetation and litter (Schmid et al. 2013) through which LiDAR cannot fully penetrate to the ground. As a result, LiDAR can overestimate elevations in vegetated settings as much as 1 m (Chassereau et al. 2011).

There is also uncertainty in datums used to calculate Z* which originate both from the datums themselves and from the extrapolation process. In short, datums encompassing more data points (higher frequency or a longer time period) have less uncertainty than datums encompassing shorter time periods; areas located further away from tide gauges have higher uncertainty than areas closer to tide gauges. While VDATUM is a useful tool for applying uncertainty through transformations, its extrapolation methodologies generate substantial uncertainty far from tidal stations (Defne et al. 2020) and the products do not extend explicitly into tidal wetlands (Brophy et al. 2019). Holmquist et al. (2018a) used empirical Bayesian kriging to extrapolate water levels and errors and calculate a probabilistic map of areas falling below the highest monthly tides. Estimating water surface elevations away from measured locations is difficult (due to surface and subsurface flow conditions). Without hydrologic restrictions, elevation maps yield a liberal estimate of flood extent and depth (Chust et al. 2008).

Despite these difficulties, we propose that a CONUS scale estimate of relative elevation can move modeling and accounting efforts forward by providing a synoptic assessment of the distribution of relative elevations, provided that the magnitude and sources of uncertainty are well documented. As far as we know, no one has calculated a national-scale Z* map, nor propagated uncertainty for Z* across a wide scale. In this paper, we present a relative tidal elevation map for CONUS tidal wetlands, generated through a transparent process, and accompanied by a corresponding uncertainty map at 30 × 30-m scale. In addition to providing these maps to encourage validation and model development, we use these maps to estimate the relative elevation of tidal wetlands as above or below the MHW line and thus representative of high- and low-elevation zones within estuarine emergent wetlands. Finally, we analyze geographic patterns in the distribution of mapped Z* and Z* uncertainty aggregated by intermediate watershed unit per the U.S. Environmental Protection Agency Hydrologic Unit framework.

We hypothesized that median tidal elevation, and variability in tidal elevation would increase from south to north. We reasoned that general resilience, and thus marsh platform building, increases with tidal amplitude (Holmquist et al. 2021a). We also reasoned that a wider tidal amplitude would lead to more variability in relative elevation. Finally, we hypothesized that propagated uncertainty in Z* would increase from north to south, because of decreases in tidal amplitude. Our focus was not on defining ecological boundaries of high marsh and low marsh vegetation species (e.g., Sanderson et al. 2001), but a framework for future analyses of the physical role of relative elevation and tidal amplitude in modeled distributions.

## Methods

While the formula for normalizing elevation can vary depending on the goal of the researcher, in our study, we calculate it as a function of orthometric elevation (*Z*) referenced to the North American Vertical Datum of 1988 (NAVD88), as well as tidal datum MHW and mean sea level (MSL) and refer to it throughout as Z*_MHW_ (Eq. 1). We make this qualification because MEM (Morris et al. 2002) references Z* relative to mean high water (MHW) and WARMER references Z* relative to mean higher high water (MHHW) (Swanson et al. 2014). We chose this formulation to differentiate wetlands that flood twice a day from those that flood between once a day and a few times a month, given mixed and semi-diurnal tides, providing a convenient and physically relevant differentiation between high-elevation and low-elevation marshes.

1 $${Z}_{\mathrm{MHW}}^{*}=\frac{Z-\mathrm{MSL}}{\mathrm{MHW}-\mathrm{MSL}}$$

### Determining Area of Interest

To create an area of interest for the Z*_MHW_ mapping, we first made key updates to the coastal lands layer presented by Holmquist et al. (2018a), a probabilistic map of areas below mean higher high water spring tide elevation (MHHWS). The updates incorporated new underlying data sources and revisions to the original methodology (Table 1). Within this updated layer, we analyzed all areas identified as estuarine wetlands according to the Coastal Change Analysis Program (C-CAP; NOAA 2014). For C-CAP palustrine wetlands, also known as freshwater wetlands, we included them in the analysis if they had greater than a 1% probability of being below MHHWS. In anticipation of users wanting to compare the relative tidal elevations of wetlands to adjacent surfaces that may represent drained, dredged, or developed former wetlands, we included farmed, developed, bare, and natural lands with the same inclusion criteria applied to freshwater wetlands. To broaden the utility of the analysis for a wide variety of users, we additionally included any areas mapped as tidal wetlands according to the National Wetlands Inventory (U.S. Fish and Wildlife Service 2014).

### Elevation and Water Level Mapping

For the analyses, we compiled coastal LiDAR-based DEMs from multiple sources, with the goal of geographic completeness. The majority of the files were aggregated for the National Oceanic and Atmospheric Association (NOAA) Sea Level Rise Viewer (NOAA 2017a). The aggregated DEMs distributed by the sea-level rise viewer, however, are not representative of all the data used in the sea-level rise viewer or the extent of historically tidal wetlands (Table S1). We additionally aggregated DEMs from the Northern Gulf of Mexico; the Sacramento Delta in California; Baltimore, and Calvert Counties in Maryland; Beaufort, and Georgetown Counties in South Carolina; Liberty and Glynn Counties, Georgia; and Mobile County, Alabama (Table S1). Our goal in selecting these DEMs was to utilize the highest quality available large-scale syntheses available, not necessarily the most up-to-date or high-quality elevation maps at the scale of individual sites, such as those available incrementally through U.S. Geological Survey’s Coastal National Elevation Database (CoNED; Danielson et al. 2018).

The majority of LiDAR DEMs have been hydro-flattened, meaning the elevation of mapped surface water was arbitrarily assigned a low number. Because the resolution of the underlying DEMs is upscaled in our processing, hydro-flattened pixels have the potential to bias surfaces, eliminating features such as berms at marsh edges. We documented hydro flattened values from file meta-data, or from a thorough inspection of the products, and masked those values from the upscaling. While minimum binning is one approach for upscaling DEMs (Schmid et al. 2011), we opted for an unbinned continuous product (Table S1). To be conservative, we also made the decision to exclude mapped water features from this analysis, including C-CAP pixels mapped as submerged vegetation and open water, in addition to LiDAR pixels that had been hydro-flattened.

For water levels, MSL, MHW, and MHHW relative to NAVD88, values came from NOAA’s (2017b) reported tidal datums. Typically, datum periods span 1983 to 2001, but some gauges with locally high rates of RSLR report datums over shorter time periods. Statistical uncertainty came from NOAA datum error reports (NOAA 2017c, 2017d, 2017e). MHHWS was a customized datum calculated relative to MHHWS from NOAA high-low data (NOAA 2016), with standard error reported, in Holmquist et al. (2018a). We used Empirical Bayesian Kriging (Krivoruchko 2012) and ArcGIS Pro 10.2 (Esri Inc. 2017) to extrapolate tidal datums from tide gauges. We used inverse distance weighting to extrapolate errors in tidal datums. See supplemental methods for additional details.

### Tidal Elevation Uncertainty Propagation

For elevation mapping, we accounted for both the bias and random error associated with LiDAR-based DEMs using a literature review (Hladik et al. 2013; Medeiros et al. 2015; Buffington et al. 2016; Holmquist et al. 2021b). In our national scale analysis, we bias-corrected using a weighted site-level average offset of 0.173 m, with a site-level weighted standard error of 0.110 m (*n* = 20 sites, 19,762 data points), and a weighted random error of 0.205 m. The propagated error at the pixel level is the sum of squares of these two uncertainties (Eq. 2), and we assumed average total *Z* uncertainty of 0.233 m.

2 $${\sigma }_{Z}^{2}={\sigma }_{\mathrm{random}}^{2}+{\sigma }_{\mathrm{bias}}^{2}$$

In this update of the probabilistic coastal lands map, in addition to propagating the uncertainty in LiDAR-based elevation, we also accounted for uncertainty between the two tidal datum transformation layers (Eq. 2).

3 $${\sigma }_{\mathrm{datum},c}^{2}={\sigma }_{\mathrm{datum},a}^{2}+{\sigma }_{\mathrm{datum},b}^{2} +2\rho {\sigma }_{\mathrm{datum},a}^{2}{\sigma }_{\mathrm{datum},b}^{2}$$

In which *ρ* represents the correlation coefficient between MHHWS relative to MHHW and MHHW relative to NAVD88, which we calculated to be 0.716.

For each tidal datum, as in Holmquist et al. (2018a), we propagated uncertainty from both the uncertainty arising from datum quality, as well as uncertainty in the extrapolation process. For the datum itself, we used the standard error according to the NOAA datum report, extrapolated using inverse distance weighting. The kriging uncertainty was sourced from the standard error of prediction from empirical Bayesian kriging. We assumed that these errors were independent, since datum error is a function of the proportion of the tidal datum period for which there is water level data at the gauge, and kriging error is a function of distance from gauge.

$${\sigma }_{\mathrm{datum},\mathrm{total}}^{2}={\sigma }_{\mathrm{datum}}^{2}+{\sigma }_{\mathrm{kriging}}^{2}$$

We applied the generalized form of an uncertainty propagation equation to the formula for Z*, resulting in Eq. 5. In this equation, *σ*_z*_ is the propagated standard deviation of the dimensionless tidal elevation map. *σ*_z_, *σ*_mhw_, and *σ*_msl_ are the standard deviations of surface elevation, MHW, and MSL, respectively.

Terms with the form ∂/∂x are partial derivatives, scalers quantifying how sensitive Z* is to variations in inputs. ∂Z*/∂*Z*, ∂Z*/∂MHW, and ∂Z*/∂MSL are the partial derivatives of elevation, MHW, and MSL, respectively. The first three terms propagate uncertainty by multiplying a sensitivity by a variance.

5 $$\begin{aligned}{\sigma }_{Z*}^{2}= & {(\frac{\partial Z*}{\partial Z})}^{2}{\sigma }_{Z}^{2}+{(\frac{\partial Z*}{\partial \mathrm{MHW}})}^{2}{\sigma }_{\mathrm{mhw}}^{2}+ {(\frac{\partial Z*}{\partial \mathrm{MSL}})}^{2}{\sigma }_{\mathrm{msl}}^{2} \\ & +2\frac{\partial Z*}{\partial \mathrm{MHW}}\frac{\partial Z*}{\partial \mathrm{MSL}}{{\sigma }_{\mathrm{mhw}}{\sigma }_{\mathrm{msl}}\rho }_{2}\end{aligned}$$

The final term propagates uncertainty arising from covariance between terms MHW and MSL. We assumed that *Z* is statistically independent of MHW and MSL, and therefore, we modeled no covariance between those terms. However, MHW and MSL were measured and interpolated from the same tide gauges and we expected them to co-vary. *ρ*_2_ is the correlation coefficient between MHW and MSL, which we calculated to be 0.873.

We calculated partial derivatives for the uncertainty propagation using the R package *Deriv* (Clausen and Sokol 2018).

6 $$\frac{\partial {Z}^{*}}{\partial Z}=\frac{1}{\mathrm{MHW}-\mathrm{MSL}}$$

7 $$\frac{\partial {Z}^{*}}{\partial \mathrm{MHW}}=\frac{Z-\mathrm{MSL}}{{\left(\mathrm{MHW}-\mathrm{MSL}\right)}^{2}}$$

8 $$\frac{\partial {Z}^{*}}{\partial \mathrm{MSL}}=\frac{Z-\mathrm{MHW}}{{\left(\mathrm{MHW}-\mathrm{MSL}\right)}^{2}}$$

### National Mapping

In addition to the updated probabilistic MHHWS map (Table 2; Holmquist et al. 2018a), we created three additional products at the scale of the CONUS including a national scale map of Z*_MHW_ according to Eq. 1, an associated uncertainty map according to Eq. 5, and a probabilistic map of low-elevation marsh. For each product, we calculated surfaces according to Eq. 1 for 65 LiDAR DEMs. Original LiDAR DEM resolutions ranged from 1 to 10 m, but all maps were resampled to 30 m resolution, with pixel extent and coordinate systems matching C-CAP. The area of interest detailed above in “Determining Area of Interest” was used as a mask layer. We removed pixels that were artificially assigned a low number because of surface water, also known as hydro-flattened pixels to avoid artificially lowering the mapped elevation when standardizing resolution to 30 × 30 m. We mosaicked files in chronological order using the minimum date for parent products reported in the file’s meta-data or file name. We did this so that if more than one raster overlapped, the newest one would be carried through to the final layer.

**Table 2:** Summary of changes made between Holmquist et al. (2018a) workflow and our reanalysis for probability elevation is lower than mean higher high water spring (MHHWS) and elevation relative to tidal amplitude at mean high water (Z*_MHW_) propagated uncertainty

Processing step	Probabilistic MHHWS (2018)	2021 update
Spatial extrapolation of datum errors	Assumed there was spatial structure, used empirical Bayesian kriging to extrapolate datum errors between gauges	Used inverse distance weighting to extrapolate datum errors between gauges
Error in LiDAR offset at local scale	Assumed LiDAR-bias uncertainty was 0	Propagated site-scale uncertainty from using a single average vegetation correction
Covariance between MHHW and MHHWS offset	Did not incorporate covariance between MHHW and MHHWS offset in uncertainty propagation	Incorporated covariance in uncertainty propagation
Mobile County, AL	Did not include. Resulted in missing patches between two other surveys	Included Mobile County in update
Baltimore County, MD	Contained an error in converting NAVD88 ft to m	Error is corrected
Southeastern counties (Georgetown, SC; Beaufort, SC; Liberty, GA; Glynn, GA)	Did not include	Included in the update
Mask	No mask	Masked out surfaces that were water or submerged aquatic vegetation in both 2006 and 2011
Mosaicking	Mosaicked files and DEM files in no particular order	Mosaicked files so that more recent DEMs override older DEMs

Since a Z*_MHW_ value of 1 is the MHW line, we mapped both “high-” and “low-”elevation marsh conditions (above and below Z*_MHW_ = 1), given a 50% probability of inclusion in either class. In order to estimate the relative area of high and low elevation marsh, we created a version of the Z*_MHW_ map which only included wetlands classified as estuarine emergent in the 2010 C-CAP maps. We also calculated a pixel-level probability of membership in each category. We normalized the thresholds relative to the mean mapped Z*_MHW_ to its respective uncertainty layer using Eq. 9. These error-normalized Z*_MHW_ scores are referred to them as *Z*′ in Eq. 9.

9 $${Z}^{^{\prime}}=\frac{x-{Z}^{*}}{{\sigma }_{{Z}^{*}}}$$

We calculated probability of membership in the low-elevation marsh class as a function of mean *Z*′ and a threshold of 1 (Eq. 10). We converted the *Z*′ to an array, converted values to cumulative probabilities using the cumulative distribution function for a normal distribution, and the empirical response function from the *Numpy* package (NumPy Developers 2017). We saved resulting probabilistic low marsh maps with two decimal points of precision. We calculated the probability of membership in the high elevation marsh class by subtracting from 1 the probability of a pixel being a low-elevation marsh (Eqs. 10 and 11).

10 $$p\left({Z}^{*}<1\right)=f(x=1,\mu ,\sigma )$$

11 $$p\left({Z}^{*}>1\right)=1-p\left({Z}^{*}<1\right)$$

We summarized area by treating each probability class as a binomial distribution and estimating mean and standard deviation using Eq. 12 and Eq. 13.

12 $$\mu = {\sum }_{i=0}^{i=100}{n}_{i}{\phi }_{i}$$

13 $${\sigma }^{2}= {\sum }_{i=0}^{i=100}{n}_{i}{\phi }_{i}(1-{\phi }_{i})$$

In which *φ* is a probability of inclusion, *i* refers to 100 0.01 wide probability class bins, and *n* is the number of pixels that fall into a probability class bin.

### Regional Summarization

We report two series of regional summary statistics. First, for lands classified as estuarine emergent wetlands according to C-CAP 2010, we summarized the area of high and low elevations by broad geographic/political regions defined by state boundaries. We classified Oregon and Washington as the Northwest; California as the Southwest; Texas and Louisiana as South Central; Mississippi, Alabama, Florida, Georgia, South Carolina, and North Carolina as the Southeast; Virginia, Maryland, the District of Columbia, and Delaware as the Mid-Atlantic; and New Jersey, Pennsylvania, New York, Connecticut, Rhode Island, Massachusetts, New Hampshire, and Maine as the Northeast.

Second, we report summary statistics for Z*_MHW_ and Z*_MHW_ uncertainty for all estuarine emergent wetlands at the scale of the intermediate watershed unit, Hydrologic Unit Code Level 8 (HUC8) (United States Department of Agriculture-Natural Resources Conservation Service [USDA-NRCS], the United States Geological Survey [USGS], and the Environmental Protection Agency [EPA] 2015). We report statistics for the subset of HUC8s which overlap mapped tidal wetlands according to the National Wetlands Inventory (NWI). For Z*_MHW_, we report mean; standard deviation; number of pixels; median; the 2.5%, 25%, 75%, and 97.5% quantiles; and the minimum and maximum values. For Z*_MHW_ uncertainty, we report HUC8-level medians.

In the course of creating these summary statistics, it became apparent that some watershed units had anomalously high median Z*_MHW_ values. We screened HUC8 watershed summaries for outliers, and omitted some from data visualization and modeling, and list them separately under Supplementary Material (2. Additional Results). We defined outliers as any watershed with a median Z*_MHW_ value greater than the 75% quantile plus 1.5 times the interquartile range.

Initial data visualization showed spatial patterns of Z*_MHW_ and Z*_MHW_ uncertainty within the coasts that we hypothesized were related to local patterns in RSLR and tidal amplitude. In order to test these hypotheses, we created corresponding RSLR and tidal amplitude maps and reported these as HUC8-level summaries. For RSLR, we queried monthly mean sea level data from any NOAA tide gauge listed as a long-term tide gauge by the Permanent Service for Mean Sea Level (Permanent Service for Mean Sea Level 2016). We chose gauges with at least 66% complete data between 1983 and 2001. We downloaded NOAA data using the R package *downloader* (Chang 2015) and calculated RSLR as the slope of a linear regression with fractional year as the independent variable and water-level in millimeters relative to station datum, as the dependent variable.

We extrapolated between gauges using empirical Bayesian kriging in ArcGIS pro (ESRI Inc 2017) using the same parameters used for extrapolating water levels (Supplemental Information). For tidal amplitude, we simply subtracted the MSL from the MHW levels kriged for the creation of Z*_MHW_ and resampled the resolution to 30 m to match the Z*_MHW_ and area-of-interest rasters. For both these layers, we summarized the median values of the rasters for each HUC8 watershed unit.

We tested hypotheses about correlations between these potential physical drivers, Z*_MHW_ properties, and general spatial trends by (1) using linear modeling and model selection techniques to create a covariate model and (2) using semi-variograms and ordinary kriging of the covariate model residuals to create a spatial model. We tested the hypothesis that watershed median Z*_MHW_ and Z*_MHW_ variability (quantified with interquartile range) were significantly correlated with watershed median RSLR and tidal amplitude, and that those two independent variables interacted with each other. For Z*_MHW_ uncertainty, we performed a simple linear regression in which Z*_MHW_ uncertainty was the dependent variable and tidal amplitude the independent variable. All dependent and independent variables were plotted with histograms to visually inspect the assumptions that distributions were normal. We natural log-transformed tidal amplitude, Z*_MHW_ variability, and Z*_MHW_ uncertainty, so that they would meet the assumptions of normality.

We used the *dredge* function in the R package *MuMln* (Bartoń 2013) in order to intercompare each possible combination of these dependent variables and determine which model structure had the optimal tradeoff between explanatory power and parsimony according to Aikake’s Information Criterion for small sample sizes (AICc). For each model, we used *anova_stats* function in the *sjstats* R package (Lüdecke 2018) to estimate effect sizes for each parameter (*ω*^2^). We hypothesized that the median Z*_MHW_ and the variability of Z*_MHW_ within watersheds would increase with tidal amplitude.

Finally, we wanted to know how other potential drivers with spatial components, such as geomorphic dynamics or the spatial autocorrelation in LiDAR errors, were affecting mapped Z*_MHW_ properties, so we fit a spatial model to the residuals of the process model. We fit semi-variograms to the residuals of each model using the R package *gstats* (Pebesma 2004; Gräler et al. 2016). In order to estimate a “pseudo-R^2^” value, we used a bootstrapping technique, leaving out one watershed at a time, fitting a semi-variogram to the rest of the watersheds, and using ordinary kriging to make a prediction for the left-out watershed. For each iteration, (i) we calculated both the error of the prediction (*x*_*i*_ − *y*_*i*_) and the error relative to the mean of the calibration dataset (*x*_*i*_ − *y*-bar). The variance of the residual model is the result of Eq. 14. The total variance explained is the adjusted (1 − *R*^2^) value from the covariate model multiplied by the residual pseudo-*R*^2^ from the spatial model.

14 $$\mathrm{Residual \;psuedo}\; {R}^{2}=1- \frac{\sum {({x}_{i}-{y}_{i})}^{2}}{\sum {({x}_{i}-\overline{y })}^{2}}$$

### Comparison to a Ground-Based Latitudinal Survey

During the course of our analyses, two of our original hypotheses, positive correlation between tidal amplitude and watershed median Z*_MHW_, and positive correlation between tidal amplitude and variability in Z*_MHW_, were refuted. The data supported alternative hypotheses at this watershed scale, namely, negative correlations between median Z*_MHW_ and tidal amplitude, and negative correlation between Z*_MHW_ variability and tidal amplitude. Because one possible explanation for this could have been artifacts arising from the GIS processing, we compared the trends in Z*_MHW_ from the LiDAR based maps to a latitudinal survey of 12 sites along the US Pacific Coast by Janousek et al. (2019).

The elevation and vegetation and plant community data needed to be reprocessed so that the time frame and metrics were comparable. We reprocessed Z* using MHW as in our study instead of MHHW as in theirs. Because the surveys were from a point in time, we calculated datums according to the year of the survey. We visually matched each site to the nearest NOAA tide gauge which had water levels referenced to NAVD88 and had complete 6-min tide gauge data for the survey year. We recalculated a custom set of tidal datums for the year the survey occurred. See supplemental information (2. Additional Methods) for additional details.

Because Janousek et al. (2019) analyzed geographic patterns in plant species niche partitioning, we simplified the dataset to unique plot-level elevation measurements only and analyzed trends in total site-wide elevation distribution. We performed two simple regression models to mirror the analysis of our remotely sensed data, one in which site-wide median Z*_MHW_ was the dependent variable, and one for which Z*_MHW_ variability (quantified with interquartile range) was the dependent variable, and both for which tidal amplitude was the independent variable. We natural log-transformed tidal amplitude and Z*_MHW_ variability, so that they would meet the assumptions of normality.

Because the trends in on-the-ground median Z*_MHW_ and Z*_MHW_ variability were similar to trends seen in the mapped data, and both observations were contrary to our hypotheses, we performed a preliminary investigation of how a few other tidal properties, which may have process links to those metrics, scale with tidal amplitude along the Pacific Coast. We referenced the same 10 tide gauges as in our reanalysis of Janousek et al. (2019) data. We hypothesized that highest astronomical tide (HAT), and/or highest observed tide (HOT), normalized to tidal amplitude at MHW (HAT*_MHW_ and HOT*_MHW_) would correlate negatively with tidal amplitude. Support for this hypothesis could point to the upland-tidal wetland interface being a “cap” that limits median wetland Z*_MHW_ over broad geographic scales. We also hypothesized that diurnal high tide inequality (DHQ), in other words the average difference between MHW and MHHW, normalized by tidal amplitude at MHW (DHQ*_MHW_) would also correlate negatively with tidal amplitude. Support for this hypothesis could point to a process link between the variability of twice daily tidal elevations and the variability in Z*_MHW_. We referenced the 1983 to 2001 tidal datums from NOAA (2017b), which fit three linear regressions with HAT*_MHW_, HOT*_MHW_, and DHQ*_MHW_ as the independent variables and tidal amplitude at MHW as the dependent variable. Tidal amplitude and DHQ*_MHW_ were natural log-transformed in order to satisfy the assumption of normality.

### General Analysis Notes

We performed data analysis using the R packages *Raster* to analyze raster data (Hijmans and van Etten 2016), *sp* to for projecting spatial data (Bivand et al. 2013; Pebesma et al. 2016), and *dplyr* for analyzing tabular data (Wickham et al. 2019). Plots 2–6 and 8 were made using *ggplot* (Wickham 2016), with *GridExtra* (Auguie 2017) and *RColorBrewer* (Neuwirth 2014). Plots 2–4 and 7–8 were made using *Rnaturalearth* (South 2017), the simple features of package *sf* (Pebesma 2018), and *ggsflabel* (Yutani 2018) to create the map elements.

## Results

**Fig. 1:**
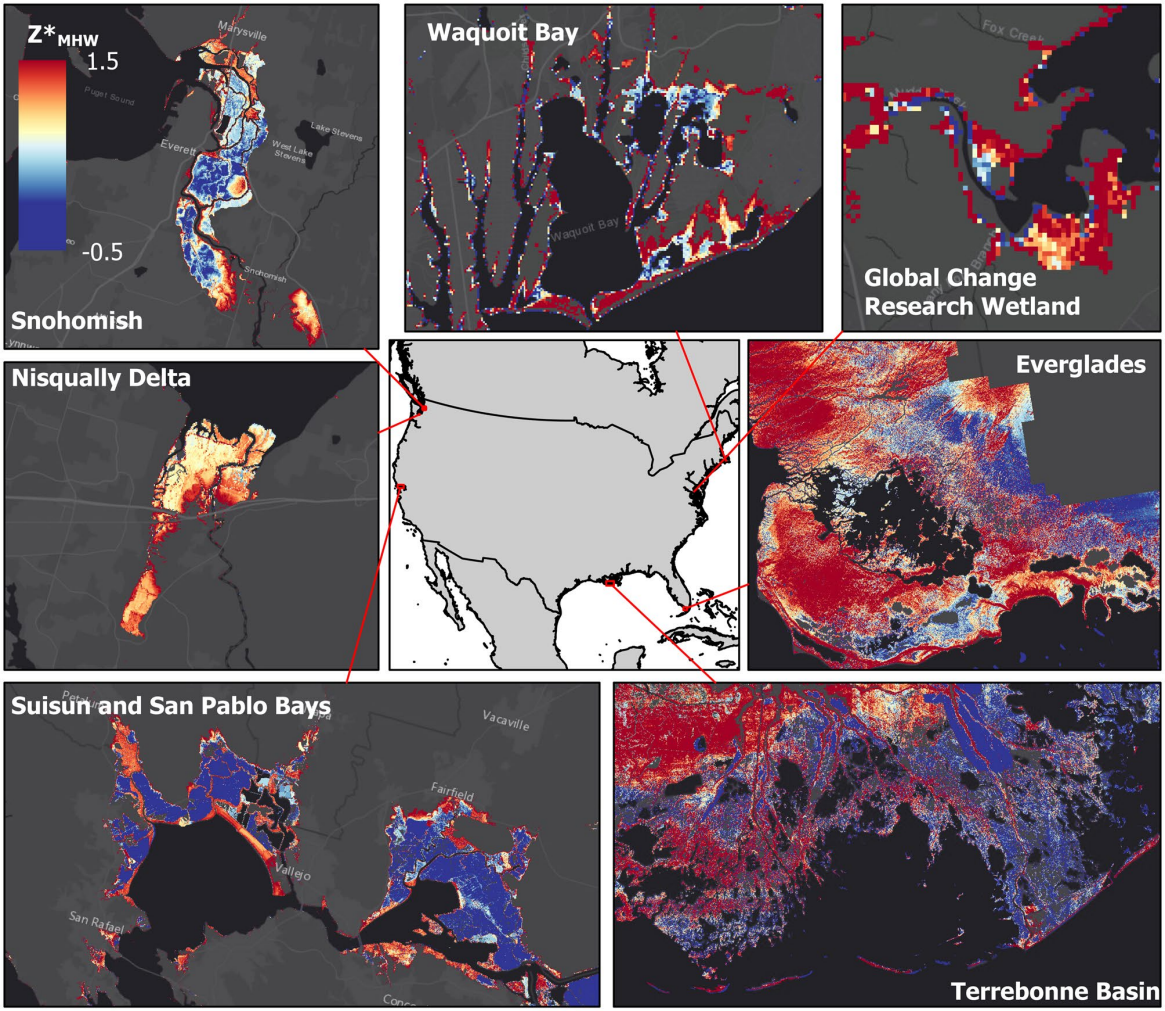
Maps of Z*_MHW_ (elevation normalized to tidal amplitude at mean high water [MHW]) representing diverse locations spanning the conterminous USA

A visualization of our Z*_MHW_ map, with a focus on six sites, is presented in Fig. 1. Additional discussion of how the map corresponds to documented elevation and land cover class observations is available under Supplemental Results.

### Area of High and Low Wetland Elevations

Of the 1.8 million hectares (M ha) of estuarine emergent tidal wetlands in the CONUS (NOAA, 2014), there are 1.18 M ha of low-elevation marsh (Table 3). This makes up 61% of estuarine emergent marshes in the CONUS. In comparison, 0.71 m ha, 39%, of estuarine emergent wetlands were classified as high-elevation marsh, receiving one tide per day or fewer given mixed and semi-diurnal tidal systems. The coverage of low-elevation marsh varies slightly across geographic/political regions of the USA ranging from 66% of marshes in the Southeast to 53% in the Northwest (Table 3).

**Table 3:** Fractional breakdown of Z*_MHW_ (elevation normalized to tidal amplitude at mean high water [MHW]) categories by region and for the entire conterminous USA (CONUS). Base data from Estuarine Emergent (EEM) Class of the 2010 Coastal Change Analysis Program. Areas are represented in hectares (ha)

Region	Mean low elevation EEM (ha)	Mean high elevation EEM (ha)	Standard error (ha)	Mean fraction % of EEM classified as low elevation (Z*_MHW_ < 1)
Northwest	5,458	4,892	9	53
Southwest	26,001	15,313	16	63
South Central	499,827	371,362	108	57
Southeast	398,903	201,265	77	66
Mid-Atlantic	98,019	72,494	49	57
Northeast	90,694	48,237	38	65
CONUS	1,118,902	713,564	147	61

### Regional Summaries

**Fig. 2:**
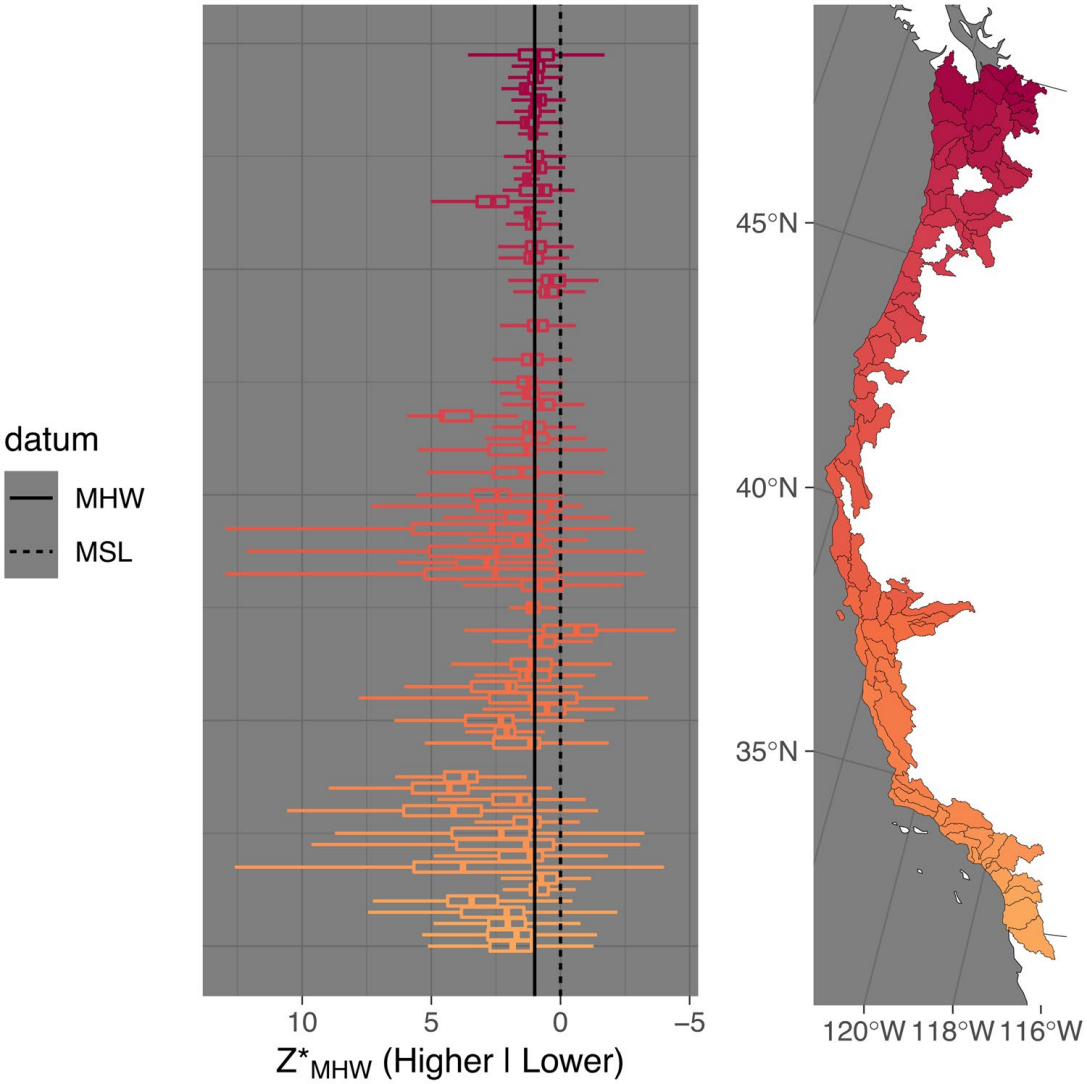
Distribution of Z*_MHW_ (elevation normalized to tidal amplitude at mean high water [MHW]) for estuarine emergent marshes of the Pacific Coast of the conterminous USA. Left panel shows the distribution of Z*_MHW_ by watershed unit arranged by latitude. The center line of the boxplot represents the median, the edges of the box the 25 and 75% quantiles, and the lines the 2.5 and 97.5% quantiles. Zero, which is mean sea level (MSL), and one, which is MHW, are plotted for reference. The right panel shows the coastal watersheds analyzed

**Fig. 3:**
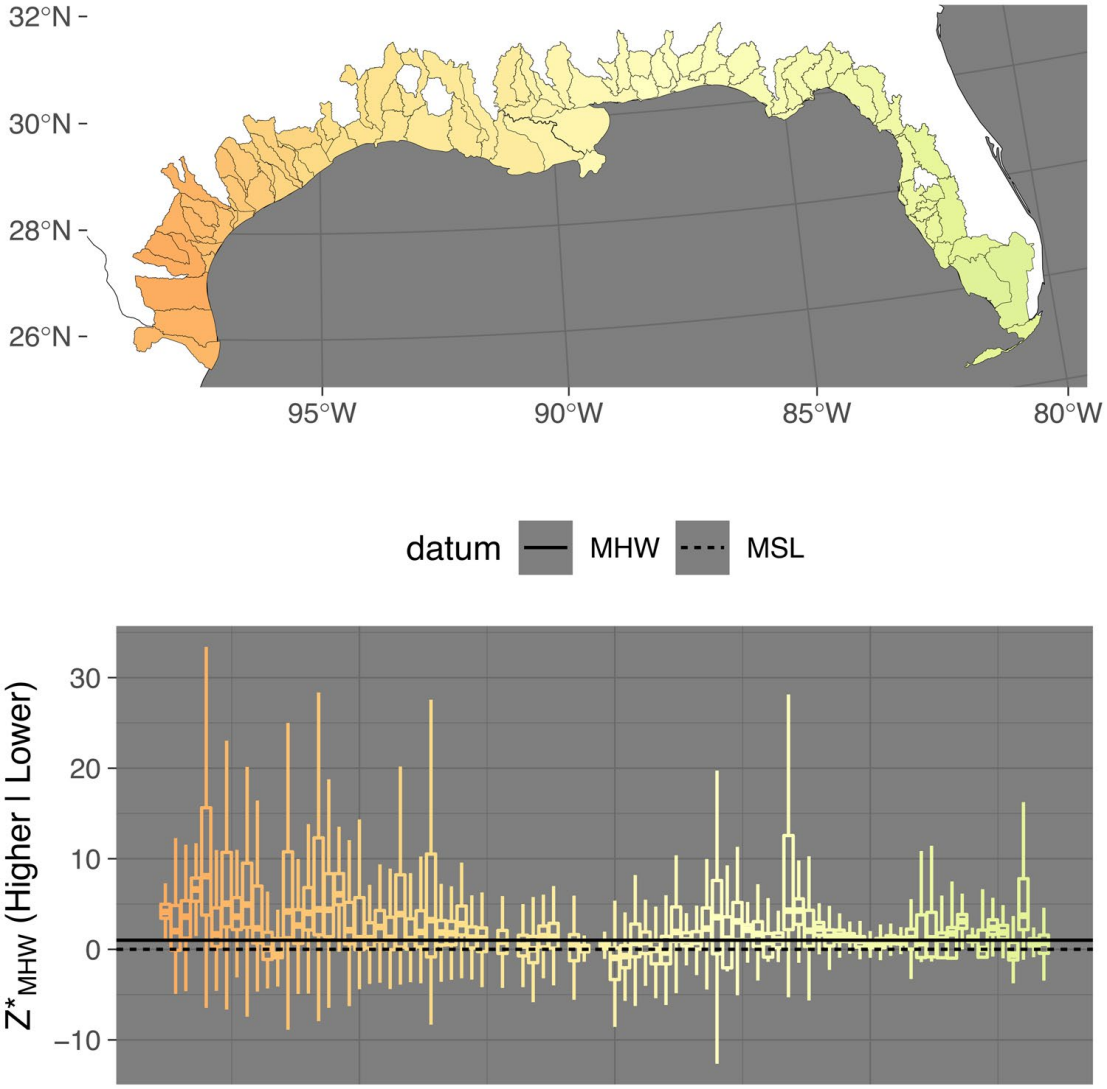
Distribution of Z*_MHW_ (elevation normalized to tidal amplitude at mean high water [MHW]) for estuarine emergent marshes of the Gulf Coast of the USA. The top panel shows the coastal watersheds analyzed. The bottom panel shows the distribution of Z*_MHW_ by watershed unit arranged by longitude. The center line of the boxplot represents the median, the edges of the box the 25 and 75% quantiles, and the lines the 2.5 and 97.5% quantiles. Zero, which is mean sea level, and one, which is mean high water, are plotted for reference

**Fig. 4:**
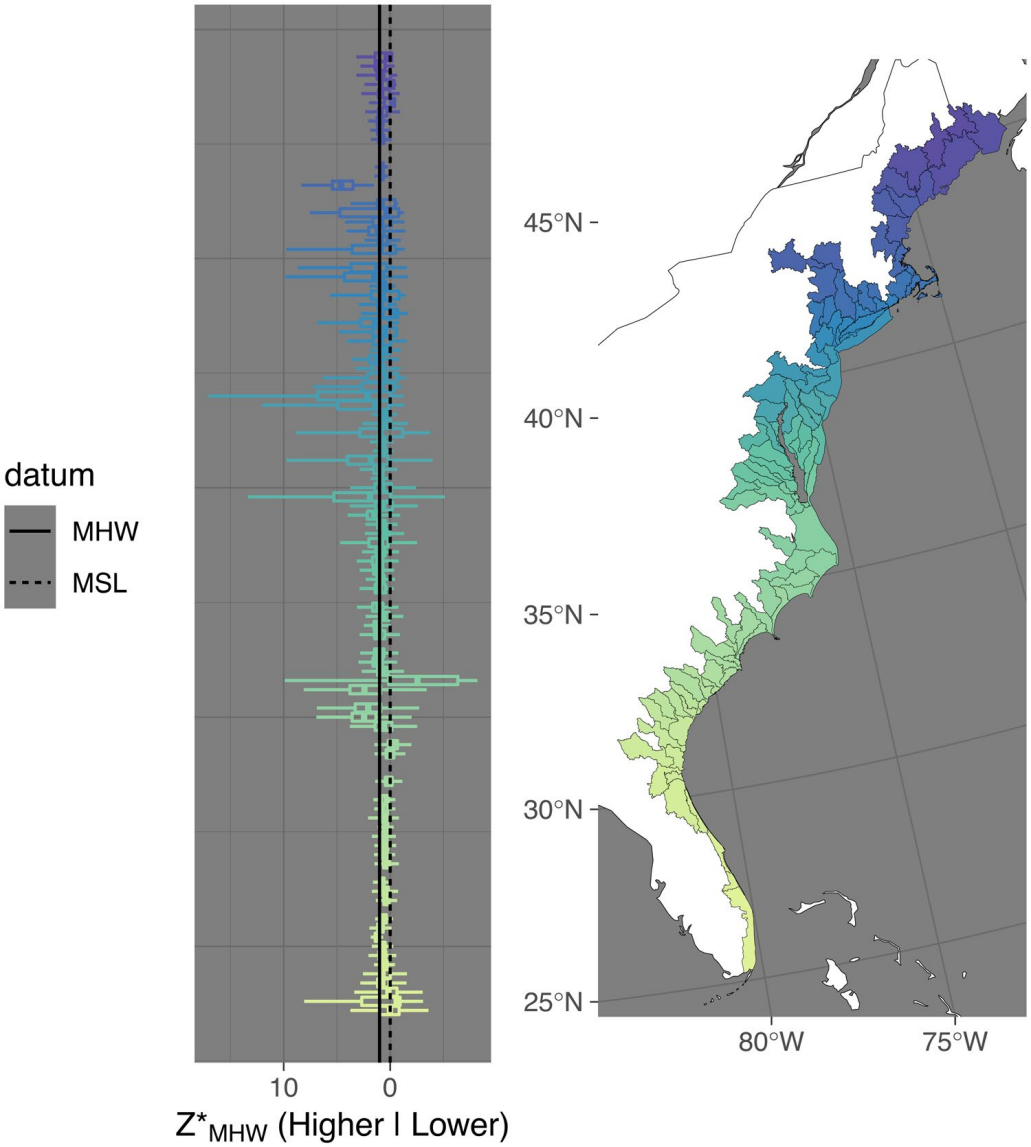
Distribution of Z*_MHW_ (elevation normalized to tidal amplitude at mean high water [MHW]) for estuarine emergent marshes of the Atlantic Coast of the USA. Left panel shows the distribution of Z*_MHW_ by watershed unit arranged by latitude. The center line of the boxplot represents the median, the edges of the box the 25 and 75% quantiles, and the lines the 2.5 and 97.5% quantiles. Zero, which is mean sea-level (MSL), and one, which is MHW, are plotted for reference. The right panel shows the coastal watersheds analyzed

**Fig. 5:**
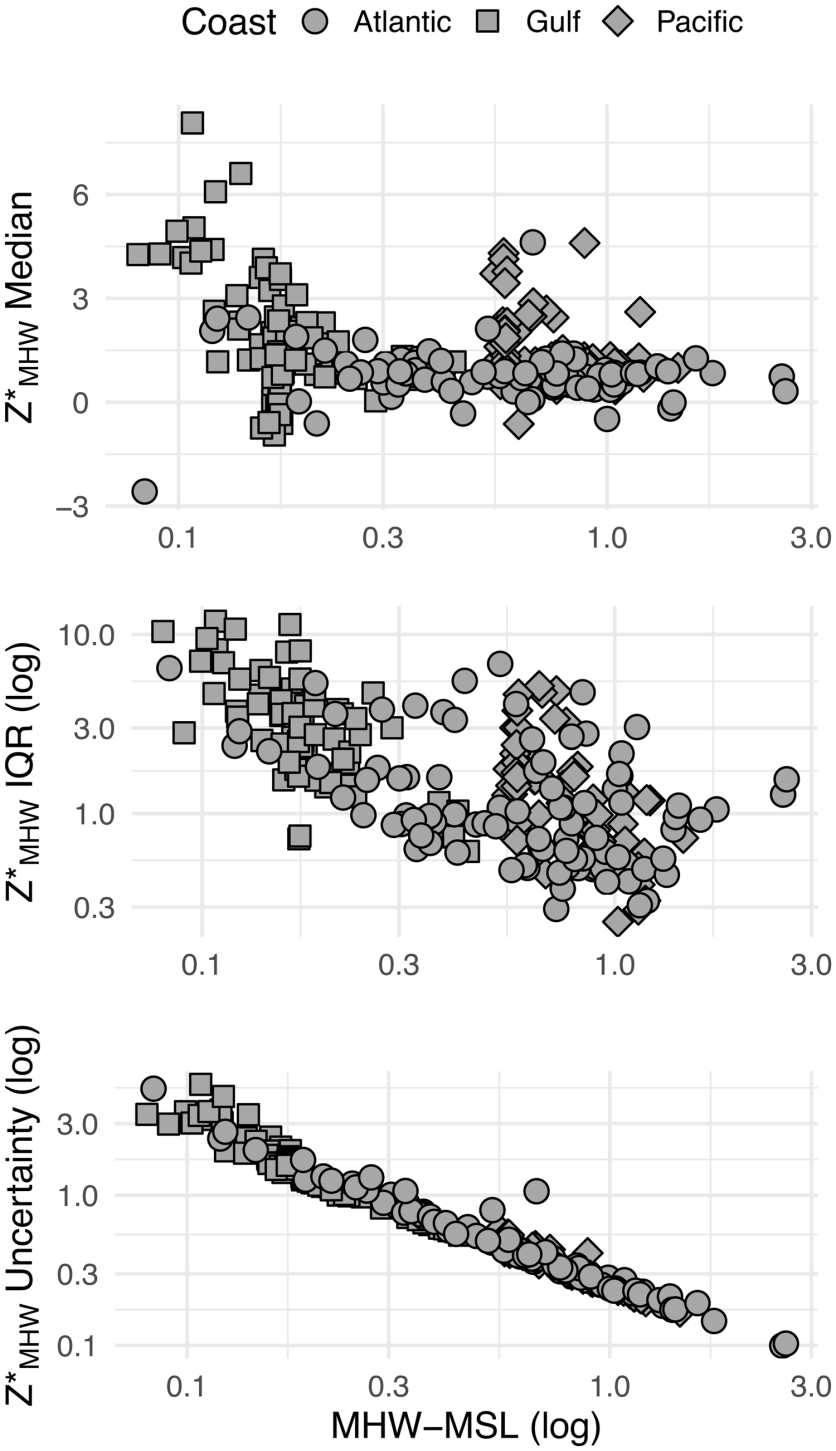
Scatterplots showing the relationship between watershed median Z*_MHW_ (elevation normalized to tidal amplitude at mean high water [MHW]) watershed Z*_MHW_ variability in the form of the interquartile range (IQR), and median watershed Z*_MHW_ uncertainty for estuarine emergent marshes, as a function of tidal amplitude (mean high water [MHW]-mean sea level [MSL]). Note that the *x*-axis and the *y*-axis of the middle and bottom plots are log_10_ transformed. MHW and MSL are in meters

The quantile distributions of Z*_MHW_ at the watershed scale shows that median elevations typically peak at a Z*_MHW_ value of 1.1 (slightly greater than MHW). Fifty percent of watersheds cluster between 0.7 and 2.0 median Z*_MHW_. Ninety-five percent of watersheds cluster between − 1.3 and 6.1 median Z*_MHW_. The median distribution of a watershed is slightly greater than 1, meaning a typical wetland classification is above MHW and thus high elevation (or infrequently flooded). Of watersheds, 55.8% had median Z*_MHW_ values that were greater than or equal to 1. This does not contradict our earlier assessment that by acreage, low-elevation marshes are the dominant CONUS marsh type. This is because a few watersheds in Louisiana contain a disproportionately high area of wetlands, and those are dominated by low-elevation marsh. The three watersheds with the most estuarine emergent wetland area are in Louisiana, they contain 21% of CONUS estuarine emergent wetland area, and they are all dominated by subtidal wetlands or low-elevation marsh: Eastern Louisiana Coastal, 8%, median Z*_MHW_ =  − 0.05; West Central Louisiana Coastal, 7.2%, Z*_MHW_ = 0.51; and East Central Louisiana Coastal, 6%, Z*_MHW_ =  − 0.95. Summary statistics for each watershed are displayed in Figs. 2–5 and listed in Table S2.

Figures 2–5 show visually that there is a high degree of spatial clustering. Watersheds that are adjacent to each other have a high degree of similarity in terms of summary statistics.

There were 12 watersheds that we classified as having median Z*_MHW_ values that were positive outliers and we include detailed observations of them under Supplemental Material (2. Additional Results). We included three outlier watersheds from Texas, but excluded the rest of the classified outliers in Figs. 2–4, and the linear and spatial modeling.

**Fig. 6:**
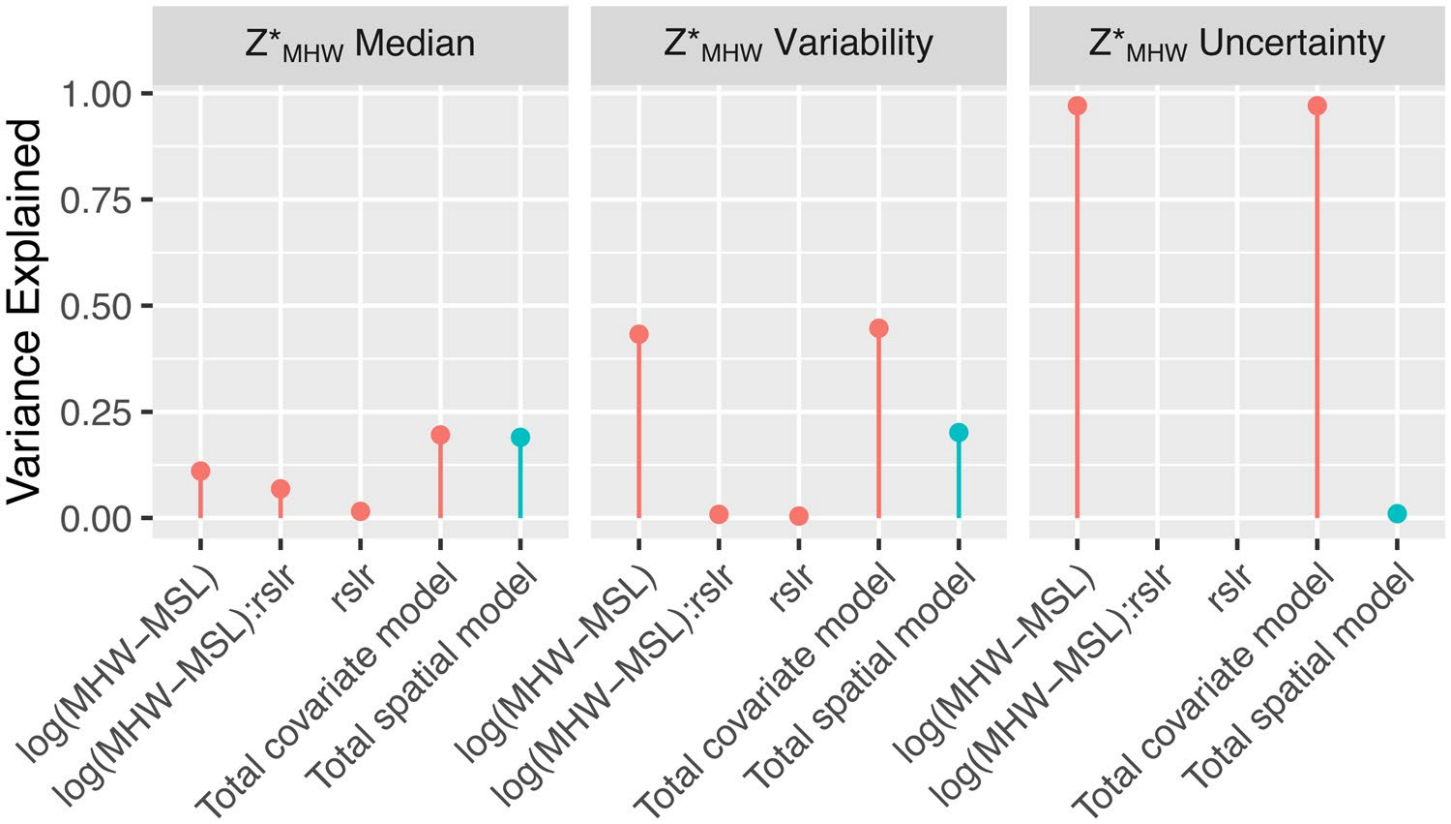
Variance explained by parameters in the covariate models (*ω*^2^), the total variance explained by the covariate models (adjusted *R*^2^), and the total variance explained by the spatial models (pseudo-*R*^2^). Red refers to the covariate model, and blue to the spatial model

For watershed-level median Z*_MHW_, the fully parameterized model had the best tradeoff between performance and parsimony. The total covariate model had an *R*^2^ of 0.20. Tidal amplitude was the most impactful parameter (Figs. 5A–C and 6), followed by the interactive effects between tidal amplitude and RSLR. The spatial model of the residuals had a pseudo-*R*^2^ of 0.19 explaining just about as much variance as the covariate model. This is visually apparent in Figs. 2, 3 and 4 in the north to south trends in median Z*_MHW_ and as well as some additional spatial clustering of median values.

The fully parameterized model predicting tidal marsh variability also had the best tradeoff between performance and parsimony. Overall, this model explained much more variance than the median Z*_MHW_ model, with an *R*^2^ of 0.45. Tidal amplitude was, by far, the most impactful parameter. The spatial model explained more variance (pseudo-*R*^2^ = 0.20) than that of median Z*_MHW_ model, but less than the covariate model for interquartile range of Z*_MHW_. The variability of marsh Z*_MHW_ is more predictable than median marsh Z*_MHW_. This is also visually apparent in Figs. 2–4 in the north to south trends in the width of the boxes, especially in the upper 25% quartile, as well as the relatively high degree of spatial clustering of IQRs.

### Comparison of Remotely Sensed Trends to Ground-Based Surveying

**Fig. 7:**
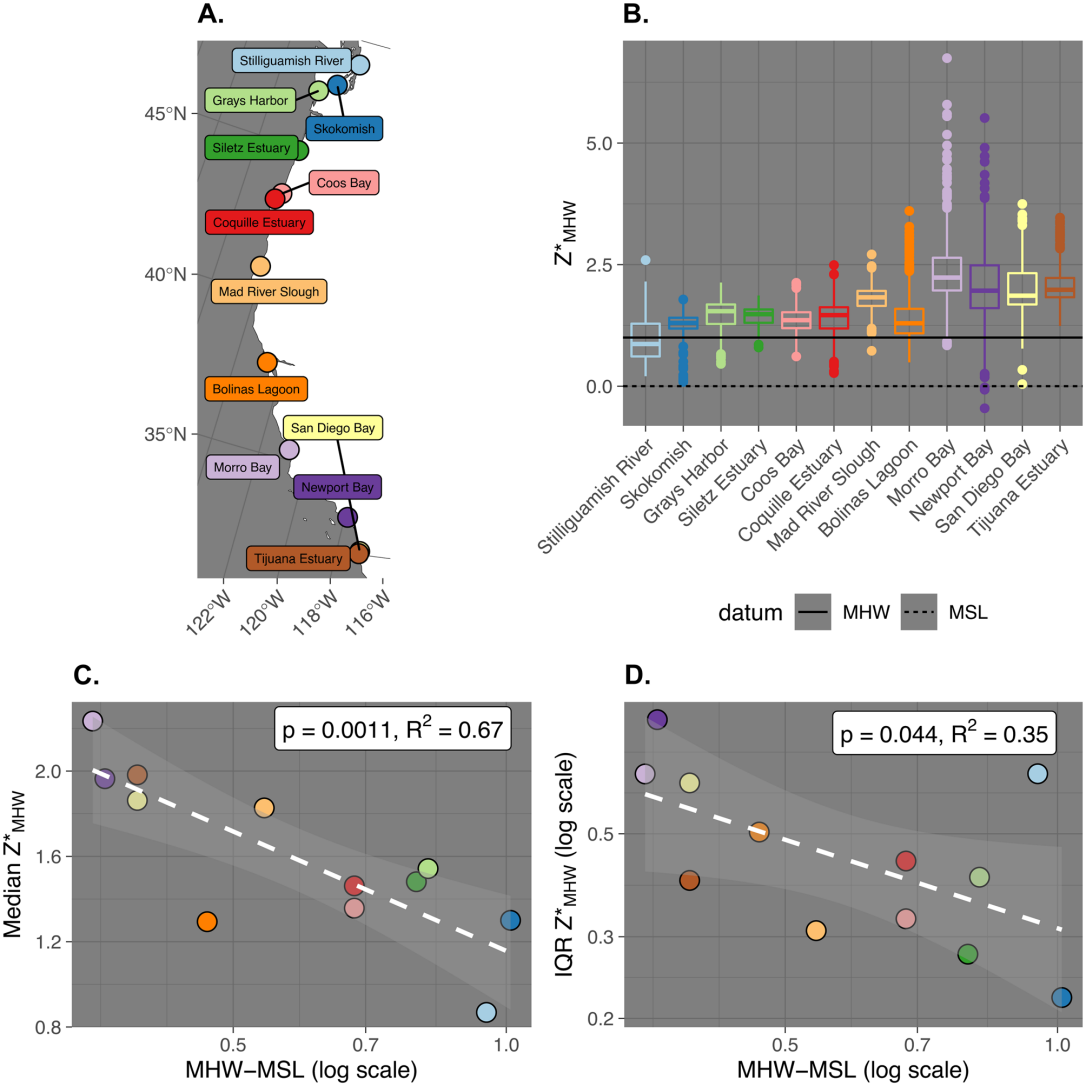
Ground-based tidal marsh elevation data from Janousek et al. (2019) reanalyzed so that Z* refers to Z*_MHW_ (elevation normalized to tidal amplitude at mean high water [MHW]). **A**. Map of the US Pacific Coast with site locations. **B** Box and whisker plots show the distribution density of the elevation distribution for individual site Z*_MHW_. The center line of the boxplot represents the median, the edges of the box the 25 and 75% quantiles, and the lines either represent the minimum, maximum, or the cutoff range for outliers (1.5 times the interquartile range). Dots represent outlier values. **C** The relationship between site-level tidal amplitude at MHW and median Z*_MHW_. **D** The relationship between site-level tidal amplitude at MHW and the interquartile range of Z*_MHW_

For the Pacific Coast analysis, we observed similar trends from our remotely sensed relative tidal elevation mapping in ground-based survey data (Janousek et al. 2019; Fig. 7). Total-site median Z*_MHW_ increased from north to south and was significantly and negatively correlated with log-transformed tidal amplitude (*p* = 0.0011, *R*^2^ = 0.67, *n* = 12). Variability in elevation generally increased from north to south as well. Log-transformed IQR of Z*_MHW_ was significantly and negatively correlated with log-transformed tidal amplitude, although the significance and variance explained was lower than the median Z*_MHW_ (*p* = 0.044, *R*^2^ = 0.35, *n* = 12).

HOT*_MHW_, HAT*_MHW_, and DHQ*_MHW_ were all significantly and negatively correlated with tidal amplitude (Fig. S1) for the 10 gauges used to calculate Z*_MHW_ for our reanalysis of the 12 sites in Janousek et al. (2019). DHQ*_MHW_ was the most strongly correlated with tidal amplitude (*p* = 6.538e − 05, R^2^ = 0.86, n = 10), followed by HAT*_MHW_ (*p* = 0.0002, *R*^2^ = 0.81, *n* = 10), and finally by HOT*_MHW_ (*p* = 0.040, *R*^2^ = 0.36, *n* = 10).

### Uncertainty in Relative Tidal Elevation

**Fig. 8:**
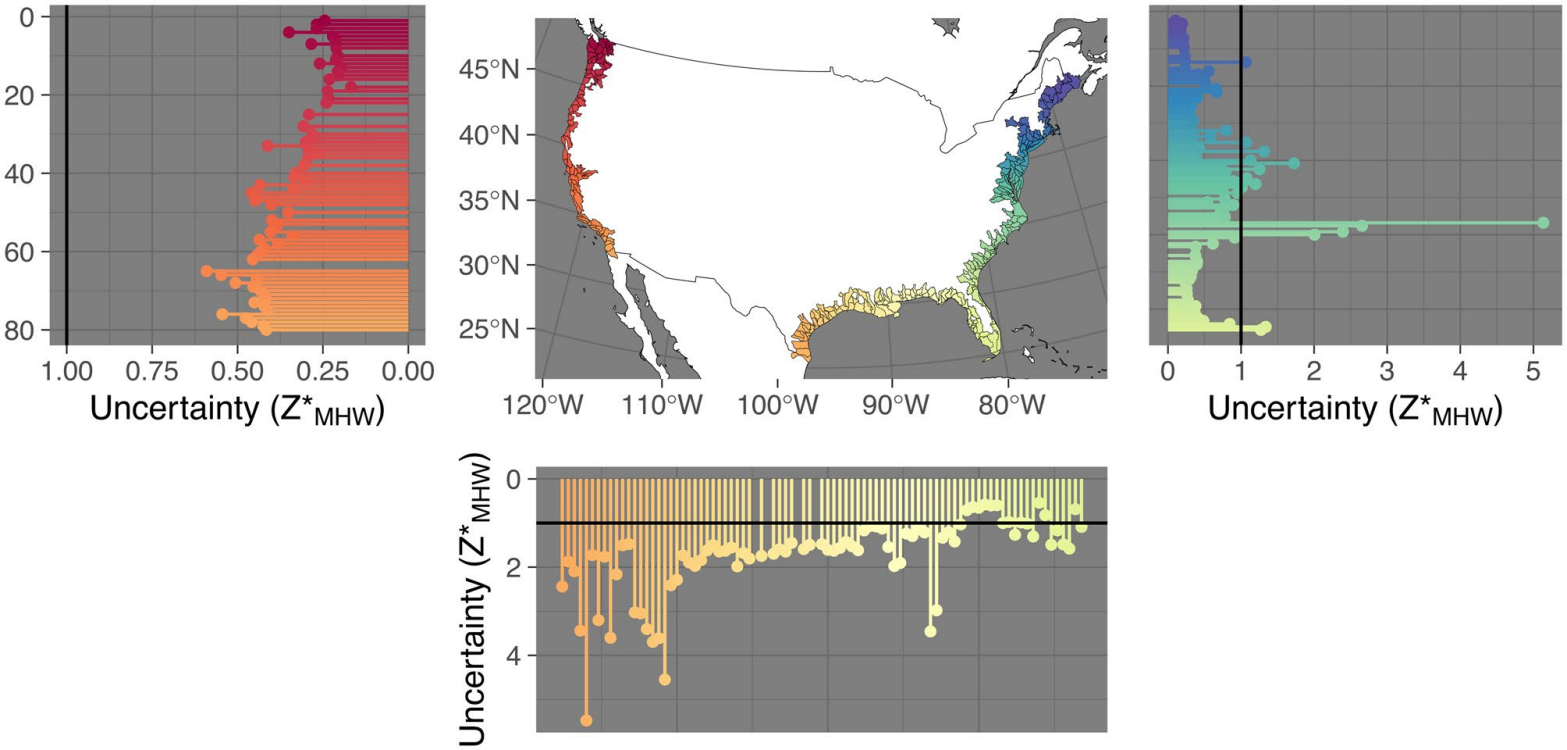
Spatial trends in watershed level Z*_MHW_ (elevation normalized to tidal amplitude at mean high water [MHW]) uncertainty for estuarine emergent marshes. Note that on the Gulf Coast, the median uncertainty in Z*_MHW_ exceeds the tidal amplitude (Z*_MHW_ = 1)

At the watershed scale, uncertainty in Z*_MHW_ was correlated significantly with tidal amplitude (*R*^2^ = 0.97, *p* < 0.0001; Table 4; Figs. 5 and 6). Watershed-level uncertainty displays spatial patterns and latitudinal gradients (Fig. 8). On the Pacific Coast, uncertainty was generally less than the difference between MHW and MSL. On the Gulf Coast, uncertainty was extreme as it was mostly greater than the tidal amplitude. For 88% of CONUS mapped estuarine emergent wetlands, LiDAR uncertainty was the dominant source of Z*_MHW_ uncertainty.

**Table 4:** Parameter summaries from covariate models. *p < 0.01, **p < 0.001, ***p < 0.0001. IQR, interquartile range; MHW, mean high water; MSL, mean sea level, *RSLR*, relative sea-level rise. MHW and MSL are in meters. RSLR is in millimeters per year

Dependent variable	Intercept	Log(MHW-MSL)	RSLR	Log(MHW-MSL) × RSLR	Adj. *R*^2^	*p*-value
Z*_MHW_ median	1.57 ± 0.17***	0.20 ± 0.22	− 0.43 ± 0.09***	− 0.38 ± 0.08***	0.20	7.326e − 12
Log(IQR Z*_MHW_)	0.01 ± 0.08	− 0.53 ± 0.12***	− 0.12 ± 0.04**	− 0.09 ± 0.05*	0.45	< 2.2e − 16
Log(Z*_MHW_ uncertainty)	− 1.37 ± 0.01***	− 1.07 ± 0.01***	NA	NA	0.97	< 2.2e − 16

## Discussion

Our goal in this paper was to share a CONUS scale product, detail its transparent production and uncertainty propagation, and examine the patterns apparent at the national scale. We found that by area, low-elevation wetlands (Z*_MHW_ < 1) are the dominant marsh type by approximately 2 to 1. However, this trend is driven primarily by a few watersheds in Louisiana which are dominated by low-elevation and subtidal wetlands and contain about 20% of the estuarine emergent marshes in the CONUS. When breaking relative percentages of high and low elevation zones down by intermediate watershed units, most watersheds are dominated by high-elevation tidal marshes (61%).

We analyzed watershed summaries of Z*_MHW_ looking at median Z*_MHW_, variability in Z*_MHW_, and uncertainty in Z*_MHW_. Both of the model fits, predicting median Z*_MHW_ and variability of Z*_MHW_, were highly significant (*p* < 0.0001). However, they both had limited explanatory power (*R*^2^ < 0.2), both refuted our hypothesis, and both supported the counter hypothesis, in which tidal amplitude correlates negatively with median Z*_MHW_ and the variability of Z*_MHW_. These trends were also observed in a reanalysis of Pacific Coast, ground-based data by Janousek et al. (2019). This gives us confidence that the trends we see are not artifactual, but are representative of real trends.

We hypothesize that the correlations we see between tidal amplitude and median Z*_MHW_ and Z*_MHW_ variability are a case of correlation not equaling causation. In other words, it could be that median elevation and/or variability in elevation is driven by phenomena that are strongly correlated with RSLR and tidal amplitude. In our limited analysis of other tidal metrics which may have more direct process relationships to median Z* and variability of Z*, we found strong correlation between tidal amplitude and HAT*_MHW_ and DHQ*_MHW_. These two metrics shrink with tidal range providing an intuitive explanation for the trends we see in our map. As tidal ranges get wider, the highest flooding elevations relative to the tidal amplitude is lower (Fig. S1). Thus, relative to the tidal amplitude, the height of the marsh-upland interface gets lower, along with the overall median Z*_MHW_ of the marsh. Likewise, the negative correlation between Z*_MHW_ variation and tidal amplitude could be explained by other tidal properties. As tidal amplitude increases, the difference between MHW and MHHW relative to the tidal amplitude at MHW decreases. Future studies could expand on the ground observations made here for the Pacific Coast and determine whether this is a more widely observable phenomenon.

The fact that median Z*_MHW_ is correlated with tidal amplitude and may be driven more directly by HAT*_MHW_ provides an important observation on which we could base further inquiry. Janousek et al. (2019), for example, use the HAT as a proxy for the marsh-upland interface. Given the lack of a physiological upper growing elevation of high marsh species, in controlled mesocosm experiments (e.g., *Spartina patens*; Langley et al. 2013; Kirwan and Guntenspergen 2015), but an upper growing limit and parabolic responses between Z* and biomass production in popular marsh elevation models (Morris et al. 2002; Swanson et al. 2014), the existence and observability of an ecological upper growing limit could be key to applying these models at scale.

Some other potential abiotic phenomena that could affect median Z*_MHW_ and its variability include lateral marsh migration space, which is driven by topography and adjacent land use (Thorne et al. 2018), and has a latitudinal trend negatively correlated with RSLR and tidal amplitude (Holmquist et al. 2021a). The lunar nodal cycle’s amplitude and phase also have spatial components (Peng et al. 2019). Potential drivers that have a latitudinal biotic component include growing degree days, temperature, and photosynthetically active radiation (Kirwan et al. 2009). In both our Z*_MHW_ models, there was a substantial proportion of the variance that could not be explained by tidal amplitude or RSLR, but could be explained by spatial autocorrelation. Some potential drivers of median Z*_MHW_ and the variability in Z*_MHW_ that could have spatial structure that is not latitudinal include (1) proximity to mineral sediment source (Weston 2014) such as a delta, or (2) storm impacts (Williams and Flanagan 2009; Morton and Barras 2011), or (3) spatial autocorrelation in LiDAR bias. The independent comparison of trends between remotely sensed and ground data (Janousek et al. 2019) gives us confidence that what we observe from remote sensing and physical water level gauges represent trends in the ecology and geomorphology of US marshes, rather than artifacts introduced by the original LiDAR data, or its post-processing.

For uncertainty in Z*_MHW_, tidal amplitude was the major driver, with the Gulf Coast watersheds almost all having uncertainties greater than one, the tidal amplitude itself. We think this brings up two questions for how these indices should be utilized and interpreted for microtidal areas. One, how practical are they to implement given the high uncertainty introduced by small tidal amplitude? Two, is Z*_MHW_ an ecologically meaningful metric in the most microtidal marshes?.

One, in our study, we found the dominant source of uncertainty in Z*_MHW_ was random error in the LiDAR-based elevation maps. We calculated uncertainty at the pixel level and did not factor in spatial autocorrelation when aggregating over larger areas. Random error should reduce as a function of the sum of squares when aggregating over larger areas and should hypothetically cancel out and approach zero when applied over larger and larger regions. The other components of uncertainty, uncertainty in LiDAR bias, and uncertainties in tidal datum transformations should be spatially autocorrelated and would not cancel out when aggregating over large areas. Future studies will need to quantify spatial autocorrelation in order to more fully account for the effect of aggregating Z*_MHW_ across different spatial scales. Overall, we think that anyone utilizing this product for derivative analyses should keep in mind that errors are likely to be more pronounced at smaller scales.

Two, this inquiry leads to whether or not Z*_MHW_ is still a meaningful ecological indicator for the most microtidal wetlands. A previous review showed that tidal amplitude is a meaningful determinant of *Spartina alterniflora* minimum and maximum elevation tolerance as tidal amplitude shrinks north to south, as observed in Mississippi and the Florida Panhandle (McKee and Patrick 1988). However, in controlled elevation mesocosm experiments performed to quantify the relationships between flooding and marsh plant productivity in the most microtidal wetlands of the USA along the Louisiana coast, researchers did not emphasize tidal datums. Instead, they compared plant growth directly to the percent of time flooded (Snedden et al. 2015; Tobias and Nyman 2017). It could be that Z*_MHW_ becomes less meaningful as a proxy for flooding as more of the variability in flooding becomes dominated by wind, river level, and storms (Snedden et al. 2015). At the very least, caution should be used when utilizing these maps at small scales in microtidal environments. At most, future studies may need to reevaluate Z*_MHW_ as a metric and flooding proxy for microtidal wetlands. It would be beneficial for researchers to have guidance on when and where applying this proxy is appropriate, and the extent to which more complex flooding-elevation profiles are needed to synoptically characterize marsh dynamics and make vegetation and carbon response predictions.

Two further directions we recommend for future researchers include continual improvement to these products, as well as their integration into vulnerability assessments and coastal monitoring programs. Leveraging existing or emerging technologies could help cover spatial gaps observed in plane-based LiDAR datasets. These other data sources include the shuttle radar topography mission (SRTM) data and space-based LiDAR, such as GEDI (Dubayah et al. 2020). Future mapping could benefit from a more fine-tuned approach to vegetation bias-corrections based on species or region or the incorporation of spatial autocorrelation in LiDAR errors. Separate water level models could likely be improved. We spatially extrapolated tidal datums using Empirical Bayesian Kriging, which neither takes into account that tidal amplitude should decrease with friction over marshes (e.g., Temmerman et al. 2005) nor takes into account water control structures and dikes. Future versions could take into account more information sources than just NOAA long-term tide gauges, (e.g., Coastwide Reference Monitoring System [CRMS] datasets, Steyer et al. 2003) and also integrate models of physical hydrology (Wu et al. 2019) to improve the predictive ability of datum maps.

In addition to continual improvements to this product, we recommend exploring its use in top-down wetland vulnerability analyses and soil carbon stocks mapping. Defne et al. (2020) showed that elevation and tidal range are tightly linked with wetland vulnerability and vegetation loss. The distribution of elevation from higher and lower marshes will be important to future mapping and forecasting of tidal wetland vulnerability to global change factors. Low marshes are more vulnerable to erosion and loss by sea-level rise, since they are already lower in the tidal frame and rely more on sediment deposition for their input (Morris and Callaway 2019). Regional surveys pairing high-quality elevation data and soil cores have demonstrated correlations between tidal elevation and carbon stocks with wetlands lower in the tidal frame tending to be more mineral dominated and wetlands higher in the tidal frame being more organic dominated (Callaway et al. 2012; Peck et al. 2020). Thus, we propose future studies investigate this link at larger scales to determine whether our Z*_MHW_ is of sufficiently high quality to map organic and inorganic-dominant soil types, thus improving coastal wetland carbon stock assessments (Holmquist et al. 2018a).

Top-down analysis of remotely sensed data can also be used to plan new or evaluate existing monitoring systems by identifying regions or zones that are over-sampled and those that are under-sampled (Shiklomanov et al. 2019). In evaluating the representativeness of surface elevation table and marker horizons used for monitoring wetland elevation change and accretion relative to relative sea-level change, Osland et al. (2017) observe that the current distribution of monitoring stations does not fully represent observed elevation gradients. They also note that for both modeling and monitoring, these observation networks need to be strategically designed to span elevation gradients as well as gradients in other relevant wetland drivers. From a modeling and monitoring perspective, there is a need to move toward quantitative mapping and reporting of surface elevation for wetland characterization. Since absolute elevation differs from tidal elevation, and tidal properties can vary regionally, we propose that our Z*_MHW_ map could be useful in other representativeness assessments for other regions needing relative and quantitative surface elevation assessments, including the Gulf Coast region. We suggest these assessments of map utility despite the high uncertainty for Z*_MHW_ metrics in the Gulf Coast region, which is unavoidable at this time given the limitations of LiDAR random error and the sensitivity of Z*_MHW_ as a metric to small tidal amplitudes.

The Coastal Carbon Research Coordination Network (CCRCN)’s data library includes survey-grade wetland elevation data if associated with sampling location of soil profiles (CCRCN 2021). However, this is only for soil coring locations. Given the potential of Z* as a proxy for tidal flooding processes, we encourage wider publication and synthesis of paired elevation survey data and contemporaneous local tidal datums (including MSL, MHW, and MHHW) for soil profiles, other types of tidal wetland monitoring such as plant cover and biomass (for example, Elsey-Quirk and Unger 2018), as well as on their own, to enable synthesis, intercomparison, and map validation.

## Conclusions

Relative tidal elevation is a vital metric for assessing coastal wetland function from a synoptic scale. Z*_MHW_ can provide a physical index of flooding exposure, or “elevation capital,” which could be an early indicator of marsh susceptibility to collapse. Z* has been correlated with soil carbon stocks, and it is part of the vital machinery of numerical marsh process models both for function and structure. With our maps, we observed latitudinal and regional trends in median Z*_MHW_ and Z*_MHW_ variability that were contrary to our hypothesis, but were supported by a reanalysis of ground-based survey data by Janousek et al. (2019). These trends may be explained by how the relative height of the marsh-upland interface and relative difference between MHW and MHHW scale with tidal amplitude. Tidal amplitude also correlates strongly with propagated uncertainty in the mapped product. Random error in LiDAR based maps is the larger source of overall uncertainty, compared to site-specific uncertainty in the bias term. Since random error in LiDAR should have little spatial autocorrelation compared to site-specific uncertainty in the bias term, overall uncertainty should decrease when used over wider scales, a testable hypothesis. Future versions of this map could be improved by LiDAR maps that have both improved random error and more sophisticated vegetation bias-corrected elevations. The Z*_MHW_ maps and underlying data used to make them are almost entirely free and available. We invite other scientists in the community to independently assess our Z*_MHW_ maps for accuracy, improve the maps through iteration, and test their utility for other even more derived purposes.

## Supplementary Information

Below is the link to the electronic supplementary material.

Supplementary file1 (CSV 42 KB)

Supplementary file2 (CSV 94 KB)

Supplementary file3 (DOCX 128 KB)

## Data Availability

Spatial data is available through Oak Ridge National Labs Distributed Active Archive Center (https://doi.org/10.3334/ORNLDAAC/1844).
