# Supplementary material for: A Conterminous USA-Scale Map of Relative Tidal Marsh Elevation
Source: Estuaries Coast. 2022 Jan 12;45(6):1596–614. doi: 10.1007/s12237-021-01027-9 (PMC9309155; doi:10.1007/s12237-021-01027-9)
Supplement: Supplementary file 3 — Supplementary file3 (DOCX 128 KB) [file 12237_2021_1027_MOESM3_ESM.docx]

**Table S1:** List of source digital elevation models (DEM) used in this analysis: demName = Internal name used for DEM; demSource = link or contact for source of the DEM; downloadDate = date downloaded (YYYY-MM-DD); hydroflatteningValue = value used to represent hydroflattened values; originalResolution = resolution of the source file in meters; SurveyYearMin = Minimum year of survey (YYYY), SurveyYearMax = Maximum year of survey (YYYY); processingNotes = Additional notes on processing and results.

**Table S2:** Watershed-level summaries of estuarine emergent relative tidal elevation and covariates used in the linear modeling exercise**.** Abbrev = Abbreviation of the hydrologic unit used in internal analysis; Name = Full name of the hydrologic unit code; States = comma-separated two-letter abbreviations for U.S. state and Canadian provinces overlapping watershed unit; Coast = One of three CONUS oceanic coasts (Pacific, Gulf, Atlantic) within which the watershed unit is located; INSIDE_X = Longitude (decimal degrees) of the watershed unit’s centroid point; INSIDE_Y = Latitude (decimal degrees) of the watershed unit’s centroid point; int_position = integer position used to order watershed units for graphing purposes; mean = watershed mean Z*_MHW_; n = number of 30 x 30 meter pixels summarized; sd = watershed Z*_MHW_ standard deviation; min = watershed minimum Z*_MHW_; Q025 = watershed Z*_MHW_, 2.5% quantile; Z*_MHW_; Q25 = watershed Z*_MHW_, 25% quantile; median = watershed median Z*_MHW_; Q75 = watershed Z*_MHW_, 75%; Q975 = watershed Z*_MHW_, 97.5%; max = watershed maximum Z*_MHW_; pct_of_all_data = proportion of total area represented by watershed; zStar_uncertainty = watershed median Z*_MHW_ propagated uncertainty; mhw_msl = tidal amplitude (meters): rslr = relative sea-level rise from 1983-2001 (millimeters per year); IQR = interquartile range of Z*_MHW_; log_MHW_MSL = Natural log of tidal amplitude; log_IQR = Natural log of Z*_MHW_ interquartile range; log_ZstarUncertainty = Natural log of propagated Z*_MHW_ uncertainty.


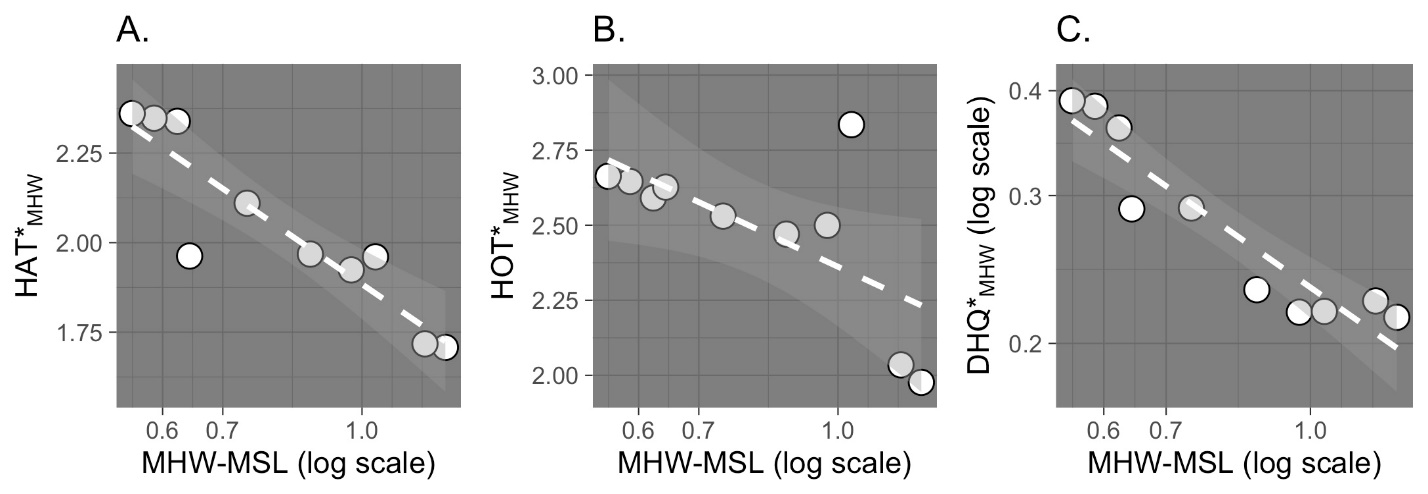


**Figure S1:** Various tidal properties normalized (*) to the tidal amplitude at mean high water (MHW) are intercorrelated with tidal amplitude at MHW itself, adding complexity to the mapped elevation trends seen in the main text and suggesting additional drivers should be considered in future work. A. the highest astronomical tide (HAT), B. highest observable tide (HOT), C. diurnal high tide inequality (DHQ), normalized (*) to the tidal amplitude at MHW, all correlate negatively and significantly with tidal amplitude at MHW. These are from 10 tide gauges used to reanalyze 12 sites worth of data from Janousek et al. (2019) for the Conterminous United States Pacific Coast. We used the tide gauges most recent, full datum period (1983 to 2001).
